# Early Brain Volume Changes After Stroke: Subgroup Analysis From the AXIS-2 Trial

**DOI:** 10.3389/fneur.2021.747343

**Published:** 2022-01-28

**Authors:** Ning Bu, Leonid Churilov, Mohamed Salah Khlif, Robin Lemmens, Anke Wouters, Jochen B. Fiebach, Angel Chamorro, E. Bernd Ringelstein, Bo Norrving, Rico Laage, Martin Grond, Guido Wilms, Amy Brodtmann, Vincent Thijs

**Affiliations:** ^1^Department of Neurology, The Second Affiliated Hospital of Xi'an Jiaotong University, Xi'an, China; ^2^Stroke Division, The Florey Institute of Neuroscience and Mental Health, Parkville, VIC, Australia; ^3^Department of Medicine and Neurology, Melbourne Brain Centre, University of Melbourne, Melbourne, VIC, Australia; ^4^Department of Medicine, University of Melbourne, Melbourne, VIC, Australia; ^5^Dementia Theme, The Florey Institute for Neuroscience and Mental Health, Parkville, VIC, Australia; ^6^Department of Neurosciences, Experimental Neurology, KU Leuven – University of Leuven, Leuven, Belgium; ^7^Laboratory of Neurobiology, VIB, Center for Brain & Disease Research, Leuven, Belgium; ^8^Department of Neurology, University Hospitals Leuven, Leuven, Belgium; ^9^Center for Stroke Research, Charité University Medicine Berlin, Berlin, Germany; ^10^Department of Neurology, University of Barcelona, Barcelona, Spain; ^11^Department of Neurology, Wilhelms University of Muenster, Münster, Germany; ^12^Section of Neurology, Department of Clinical Sciences, Lund University, Lund, Sweden; ^13^Department of Clinical Research, Guided Development GmbH, Heidelberg, Germany; ^14^Department of Neurology, Kreisklinikum Siegen, University of Marburg Germany, Marburg, Germany; ^15^Department of Radiology, University Hospitals of Leuven, Leuven, Belgium

**Keywords:** ischemic stroke, brain volume changes, atrophy, clinical trial, hemorrhagic transformation, edema

## Abstract

**Background and Purpose:**

The evolution of total brain volume early after stroke is not well understood. We investigated the associations between age and imaging features and brain volume change in the first month after stroke.

**Methods:**

We retrospectively studied patients with acute ischemic stroke enrolled in the AXIS-2 trial. Total brain volume change from hyperacute MRI data to the first month after stroke was assessed using unified segmentation in SPM12. We hypothesized that age, ischemic brain lesion size, and white matter (WM) changes were associated with larger brain volume change. Enlarged perivascular spaces (EPVSs) and white matter hyperintensities (WMHs) were rated visually and the presence of lacunes was assessed.

**Results:**

We enrolled 173 patients with a mean age of 67 ± 11 years, 44% were women. There was a median 6 ml decrease in volume (25th percentile −1 ml to 75th percentile 21 ml) over time, equivalent to a median 0.5% (interquartile range [IQR], −0.07%−1.4%), decrease in brain volume. Age was associated with larger brain volume loss (per 10 years of age, 5 ml 95% *CI* 2–8 ml). Baseline diffusion weighted imaging (DWI) lesion volume was not associated with greater volume loss per 10 ml of lesion volume, change by 0 ml (95% *CI* −0.1 to 0.1 ml). Increasing Fazekas scores of deep WMH were associated with greater tissue loss (5 ml, 95% *CI* 1–10 ml).

**Conclusions:**

Total brain volume changes in a heterogenous fashion after stroke. Volume loss occurs over 1 month after stroke and is associated with age and deep WM disease. We did not find evidence that more severe strokes lead to increased early tissue loss.

## Introduction

Cerebral atrophy is the end result of chronic or acute insults affecting the brain and commonly occurs with aging and stroke. Yearly rates of atrophy increase with age, with estimated changes of approximately −0.41% in healthy individuals (1) in their seventh decade, and rates up to −2.39% in patients with cerebral small vessel disease (2). In chronic stroke, tissue loss is obvious in the area of the stroke. In addition, there is evidence of more widespread and distant loss of brain tissue in the hippocampus (3) and other regions (4). Risk factors for the development of atrophy after stroke are poorly understood (5). On the one hand, one may hypothesize that volume changes may be more prominent in larger strokes, where tissue loss is extensive in the area of the insult. However, these larger strokes may be associated with more edema and hemorrhagic transformation, with resultant increases in volume, especially in the first few days and weeks after stroke. Cerebral small vessel disease and age are associated with accelerated brain volume changes, when patients are studied serially after stroke (5–11). Cerebral volume changes are detectable using advanced imaging techniques (8–10).

Brain volume loss is increasingly used as a surrogate outcome marker in clinical trials of interventions targeting the cognitive impairment after stroke. The time course of global brain volume change after ischemic stroke has only recently been studied in detail and focused mostly on changes occurring after 1–3 months (5).

To obtain insight into the earliest phase of brain volume changes after stroke, we estimated the size of the brain volume change between baseline and 30 days after stroke and investigated the associations between age, stroke severity, and size and presence of SVD markers, and brain volume change in the first month after stroke. This may inform the timing of future interventions targeting brain volume loss.

We additionally investigated the association between brain volume change and functional outcome, measured using the modified Rankin scale (mRS).

## Methods

We performed a *post-hoc* analysis of the AXIS-2 study, a European, multicenter, placebo-controlled, randomized, and double-blind trial of granulocyte colony-stimulating factor (G-CSF) after ischemic stroke.

Details on the AXIS-2 trial protocol, statistical analysis plan, baseline characteristics, and main results have been published previously (12). In summary, AX-200 recruited 324 patients in 51 centers in eight countries. Patients with acute ischemic stroke were randomly assigned to either placebo or G-CSF therapy [a cumulative dose of 135 μg/kg body weight recombinant human G-CSF (Filgrastim; AX200; SYGNIS, Germany) over 72 h].

### Standard Protocol, Approvals, Registrations, and Patient Consents

The study was performed according to the International Council of Harmonization Good Clinical Practice and was approved by the respective regulatory authorities. Informed consent of patients was required before they were entered into the study. Design and content of the consent form were according to the country regulations and approved by the lead and local ethics committees.

### Study Population

The main inclusion criteria were initial National Institutes of Health Stroke Scale (NIHSS) score from 6 to 22, age 18–85 years, a time window of ≤ 9 h after onset of stroke symptoms, and stroke localization in the middle cerebral artery territory. Treatment with recombinant tissue plasminogen activator (rt-PA) was allowed whenever patients fulfilled all these criteria after having received thrombolysis. The main exclusion criteria for the randomized trial were signs of a very severe stroke on imaging (carotid T occlusion, involvement of more than two-thirds of the middle cerebral artery territory, and signs of midline shift), hemorrhagic, and lacunar strokes.

Recruitment started in August 2009, and the total study duration was 24 months. Physicians who examined patients for outcome parameters were trained and certified for mRS and NIHSS evaluation. NIHSS score was obtained at baseline (day 1) and mRS scores were determined at 90 days after inclusion.

### Imaging

Patients underwent MRI, such as fluid attenuation inversion recovery (FLAIR), T2, and diffusion weighted imaging (DWI) within 9 h of onset and FLAIR and T2 images at 1 month after stroke onset. MRI readings were performed using a proprietary semiautomatic reading system (BioClinica, Lyon, France) by three independent and blinded readers. DWI and FLAIR volumes were delineated semi-automatically using proprietary segmentation and editing tools. The lesion volumes were averaged over the readers.

MRI data were obtained on 1.5T scanners in 90% of scans, from Siemens (78%), Philips Medical Systems (9%), General Electric (13%), and Toshiba (0.3%). Siemens 3T scanners were used in the remaining 10%. For the T2 images, the median number of slices was 25 (24–28), repetition time 4,000 ms (3,000–4,200), echo time 90 ms (80–97), flip angle 150 degrees (90–150), slice thickness 5 mm (5–5), interslice gap 5 mm (5–5), image matrix 256 (192–448) columns and 256 (256–512) rows, voxel size x (mm) 0.94 (0.47–0.94), voxel size y (mm) 0.94 (0.47–0.94). For the FLAIR images, the median number of slices (interquartile range [IQR]) was 25 (24–27), echo time 90 ms (80–100), flip angle 150 degrees (150–150), slice thickness 5 mm (5–5), interslice gap 5 mm (5–5), image matrix 256 (192–388) columns and 256 (256–512) rows, voxel size x (mm) 0.75 (0.47–0.94), voxel size y (mm) 0.75 (0.47–0.94).

Serial scans were obtained on the same scanner in 93% of patients. The exact same image resolution (x, y, z dimensions) was used in 75% of patients on the baseline and follow-up scan.

Deep white matter hyperintensities (DWMHs) and periventricular white matter hyperintensities (PWMHs) were assessed with the Fazekas scale ranging from 0 to 3 [DWMH: 0 = no lesions, 1 = punctate foci, 2 = beginning confluency of foci, 3 = large confluent areas; PWMH: 0 = no lesions, 1 = caps or thin line, 2 = smooth halo, 3 = extension to white matter (WM); Fazekas et al. (13)]. MRI-visible enlarged perivascular spaces (EPVSs) were defined as small (<3 mm) punctate hyperintensities on T2 images based on a previously validated scale. (14) We rated basal ganglia, centrum semiovale, and midbrain EPVS. Basal ganglia and centrum semiovale EPVSs were rated 0 (none), 1 (1–10), 2 (11–20), 3 (21–40), and 4 (>40), and midbrain EPVSs were rated 0 (none visible) or 1 (visible). At the midbrain, basal ganglia and centrum semiovale levels, we counted EPVSs on both sides and summed the EPVS scores of each level.

### Brain Volume Measurements

All brain segmentations were performed using the SPM12 (*http://www.fil.ion.ucl.ac.uk/spm/software/spm12/**)* toolkit using MATLAB R2017b (The MathWorks Inc., Natick, MA, USA). SPM12 performs automatic segmentation of T2 weighted MR-images into WM, gray matter (GM), and cerebrospinal fluid (CSF) by the use of a unified model of image registration, tissue classification, and bias correction. (15) The default settings of the automated SPM 12 segment pipeline were used. The segmented images of GM, WM, and CSF were warped to native tissue. Total brain volume was measured by adding GM, WM, and CSF volumes.

### Statistical Analysis

We used quantile regression with bootstrapped standard error estimation and 500 repetitions in STATA version 15.1 (Corporation College Station, TX, USA) to analyze variables associated with total brain volume changes over time. This method does not require assumptions regarding the distribution of brain volumes and is less influenced by outliers. We report associations with the median, the 25th and 75th quantile of the distribution of volume change. Factors in the models were prespecified and based on the existing literature on brain atrophy and outcome after stroke. We prespecified the following hypotheses: (1) brain volume change would be more pronounced with advancing age and (2) with stroke severity, measured using larger baseline ischemic lesion volumes, stroke impairment using the NIHSS and 30 d FLAIR lesion volume and (3) with the presence of markers of small vessel disease. For hypothesis 1 and 2, we tested age and baseline ischemic lesion volume on DWI, NIHSS, and 30 d FLAIR volume as the independent variables, respectively, and brain volume change as the dependent variable, adjusting for baseline brain volume. For hypothesis 3, deep WM and periventricular WM, enlarged perivascular spaces and the presence of lacunes were tested as the independent factors, adjusted for the baseline brain volume. Ordinal logistic regression was used to test whether brain volume change was associated with functional outcomes using the mRS at 90 days. The mRS of 5 and 6 were collapsed in this analysis. We adjusted for age, baseline NIHSS, and baseline DWI lesion volume. Multicollinearity between included variables was tested by the variance inflation factor. A two-tailed *p* < 0.05 was considered statistically significant. No correction for multiple comparisons was performed.

## Results

### Characteristics of the Study Population

A total of 324 patients with acute ischemic stroke were enrolled in the AXIS-2 trial. T2 MRI was lacking or of poor quality in 61 patients, 86 patients did not undergo a repeat T2 MRI and 4 patients lacking a baseline DWI lesion volume measurement were excluded. This left 173 patients for analysis in this study. These were recruited from 35 participating centers who enrolled a median of 3 (IQR 2–6, minimum 1–31) patients. The baseline age, NIHSS score, DWI lesion volume, and follow-up mRS score at 90 days were different between the patients who were included in this study and the excluded patients. The patients without follow-up MRI were older, NIHSS scores were higher, DWI lesion volumes were larger, and follow-up mRS scores at 90 days were worse. Other baseline demographic characteristics and baseline imaging markers were not significantly different between the two groups (as shown in Table 1). In the included group, there were 97 men (56%) and 76 women (44%). The mean age of the patients was 67 years (age range 40–85 years, SD 11). Baseline median NIHSS was 10 (IQR 8–13), the median baseline diffusion lesion volume median was 22 ml (IQR 8–49 ml). The follow up MRI was obtained at 31 days (IQR 29–33 days). The median FLAIR lesion volume at that time was 32 ml (IQR 10–78 ml). The median change in ischemic lesion volume between baseline and follow-up was 7 ml (IQR −0.5 to 31 ml).

**Table 1:** Comparison of baseline characteristics of patients with and without follow-up MRI.

	**Population with follow–up MRI**	**Population without follow–up MRI**	***P–*value**
N	173	86	
Age, median, (IQR)	70 (61–76)	75 (68–80)	<0.0001
Male, n (%)	97 (56%)	42 (49%)	0.272
NIHSS score median (IQR)	10 (8–13)	14 (10–18)	<0.0001
Baseline total brain volume median (IQR), mL	1,360 (1,278–1,465)	1,315 (1,242–1,447)	0.152
Global score of EPVS, median (IQR)	7 (6–9.5)	8 (6–10)	0.224
EPVS-MB, median (IQR)	1 (0–2)	1 (0–2)	0.475
EPVS-BG, median (IQR)	2 (2–4)	2 (2–4)	0.615
EPVS-CSO, median (IQR)	4 (2–6)	4 (2–6)	0.493
DWMH, median (IQR)	1 (1–2)	1 (1–2)	0.55
PWMH, median (IQR)	2 (1–3)	2 (1–3)	0.162
Lacunes, n (%)	8 (5%)	1 (1%)	0.152
DWI lesion volume, median (IQR), mL	22 (8–49)	34 (12–83)	0.008
Intravenous tPA, n (%)	117 (68%)	58 (67%)	0.976
G-CSF, n (%)	84 (49%)	45 (52%)	0.628
Follow-up mRS score at 90 days, *n*			
0	23 (13%)	9 (10%)	<0.0001
1	41 (24%)	11 (13%)	
2	27 (16%)	7 (8%)	
3	21 (12%)	5 (6%)	
4	36 (21%)	7 (8%)	
5	16 (9%)	10 (12%)	
6	9 (5%)	37 (43%)	

*Interquartile range (IQR): 25th−75th percentile*.

*NIHSS, National Institutes of Health Stroke Scale; EPVS, enlarged perivascular spaces; MB, middle brain; BG, basal ganglion; CSO, centrum semiovale; SVD, small vessel disease; DWMHs, deep white matter hyperintensities; PWMHs, periventricular white matter hyperintensities; mRS, modified Rankin scale*.

### Brain Volume Loss Over Time

The median total brain volume at baseline was 1,360 ml (IQR 1,278–1,465 ml) and 1,351 ml (IQR 1,273–1,443 ml) at follow-up. In 48 (28%) patients, total brain volume was increased with a median increased volume of 12 ml (IQR 6–27 mL) and in 125 (72%) patients, brain volume was decreased, median decreased volume was 16 ml (IQR 5–25 ml). The baseline characteristics (baseline NIHSS, baseline diffusion lesion volume, age, and sex) and outcomes measured with the mRS at 30 and 90 days of the patients who had a decrease in brain volume were not significantly different from those with an increase in brain volume. FLAIR lesion volume at 30 days (*p* = 0.263) and lesion volume change over time (*p* = 0.171) were not different between patients with an increased total brain volume and those with a decreased total brain volume. Overall, there was a median 6 ml decrease in volume (25th percentile −1 ml to 75^th^ percentile 21 ml) over time. This is equivalent to a median 0.5% (IQR −0.07% to 1.4%), decrease in brain volume. Female sex and a history of prior stroke were associated with smaller total brain volumes but were not associated with brain volume change. GM-CSF treatment was not associated with serial changes in brain volume. In addition, image resolution differences between the baseline and follow-up were not associated with brain volume change.

### Age and Brain Volume Change

**Figure 1:**
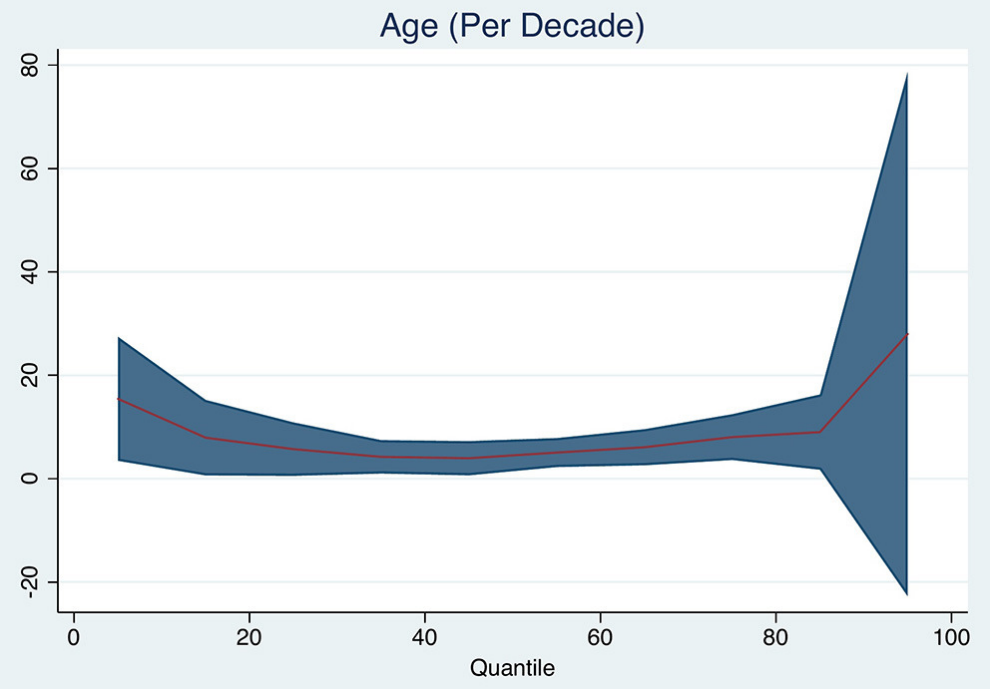
Quantile plot showing estimated brain volume change in milliliter and 95% confidence limits (*y*-axis) according to age (decades). An *x*-axis shows the quantile levels. Positive numbers in the *y*-axis reflect decrease in brain volume.

Age was associated with brain volume change (Figure 1). Brain volume was reduced by a median of 5 ml (95% *CI* 2–8 ml, *p* = 0.002, pseudo-R^2^ 0.043) for each increasing decade, adjusted for baseline total brain volume. Additionally, age was associated with the 25th quantile of brain volume change (median 6 ml, 95% *CI* 1–11 ml, *p* = 0.023, pseudo-R^2^ 0.046) and with the 75th quantile of brain volume change (median 8 ml, 95% *CI* 4–12 ml, *p* < 0.001, pseudo-R^2^ 0.037).

### Stroke Severity and Size and Brain Volume Change

**Figure 2:**
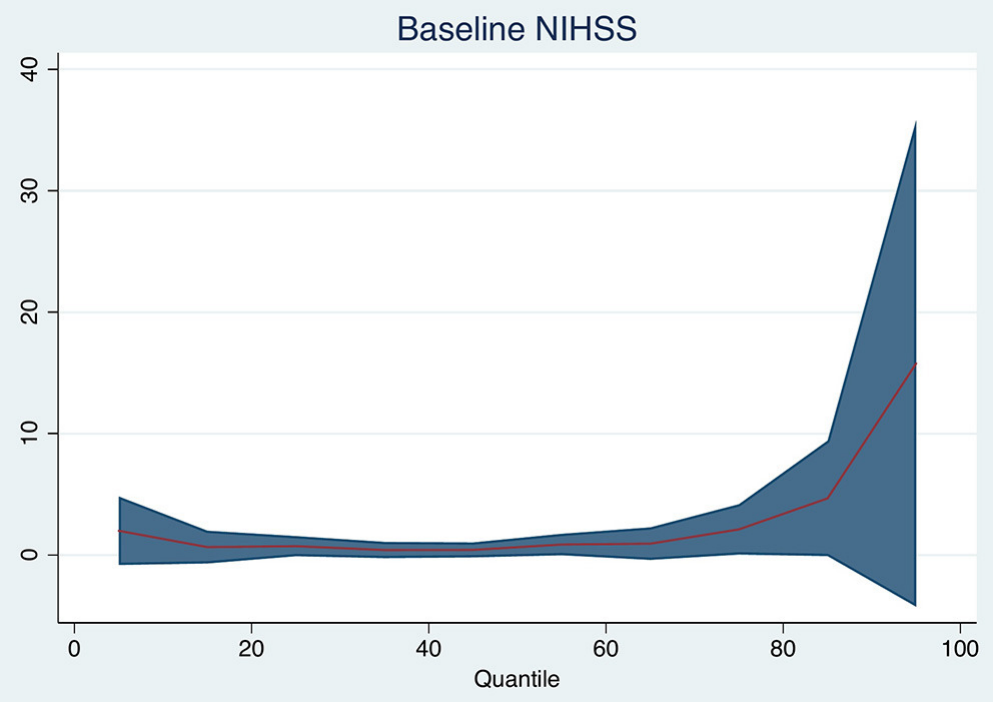
Quantile plot shows the estimated brain volume change in milliliter and 95% confidence limits (*y*-axis) according to baseline NIHSS (per point). An *x*-axis shows the quantile levels. Positive numbers in the *y*-axis reflect decrease in volume.

Baseline DWI lesion volume (per 10 ml increase in baseline DWI lesion, median 0 ml, 95% *CI* −1 to +1 ml, *p* = 0.760), 30-day FLAIR lesion volume (median 0, 95% *CI* −1 mL to 1 ml, *p* = 0.940), and baseline NIHSS (median 0, 95% *CI* 0–1 ml, *p* = 0.180) were not associated with the median change in brain volume. Table 2 and Figure 2 show the changes for the 25th and 75th quantiles of brain volume change. Baseline NIHSS was associated with the 75th quantile of brain volume change (*p* = 0.048, pseudo-R^2^ 0.031). There was no association between the change in ischemic lesion volume between baseline on DWI and 30 days on FLAIR and brain volume change (*p* = 0.908).

**Table 2:** Brain volume change and stroke severity/size, adjusted for baseline brain volume (positive numbers indicate decrease in volume).

**Variable**	**25th percentile** **(95% CI), mL**	***P*-value**	**Median** **(95% CI), mL**	***P*-value**	**75th percentile** **(95% CI), mL**	***P*-value**
NIHSS (change in volume per 1 point increase)	1 (0–2)	0.093	1 (0–1)	0.180	2 (0–4)	0.048
Baseline DWI volume (per 10 mL)	0 (−1–+1)	0.709	0 (−1–+1)	0.760	1 (−1–+3)	0.502
30day FLAIR total volume (per 10 mL)	0 (−1–+1)	0.778	0 (−1–+1)	0.940	0 (−1–+1)	0.553

*NIHSS, National Institutes of Health Stroke Scale; CI, confidence interval; and DWI, diffusion weighted imaging*.

### Small Vessel Disease and Brain Volume Change

**Figure 3:**
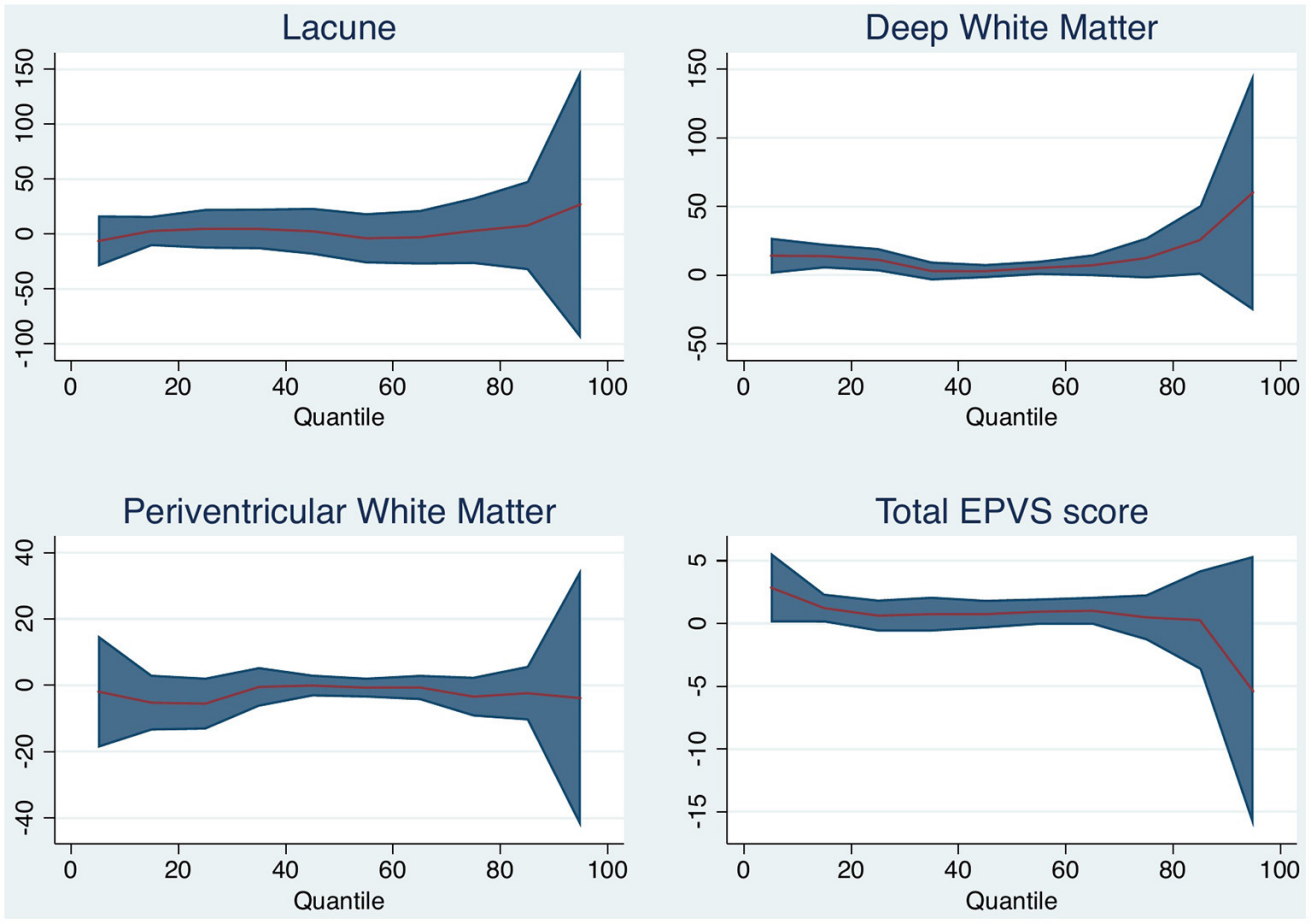
Quantile plot shows estimated brain volume change in milliliter and 95% confidence limits (*y*-axis) according to white matter (WM) disease characteristic. An *x*-axis shows the quantile levels. Positive numbers in the *y*-axis reflect decrease in brain volume.

Increasing degrees of DWMH associated with brain volume change (per point increase on the Fazekas scale, median 5 ml, 95% *CI* 1–10 ml, *p* = 0.021). DWM was associated with median brain volume change and with 25th quantile change, but not with the 75th quantile change. The other small vessel disease markers were not associated with brain volume change. Table 3 and Figure 3 shows the associations for the 25th and 75th quantiles of brain volume change.

**Table 3:** Brain volume change and small vessel disease imaging markers, adjusted for baseline brain volume (positive numbers indicate decrease in volume).

**Variable**	**25th percentile** **(95% CI), mL**	***P*-value**	**Median** **(95% CI), mL**	***P*-value**	**75th percentile** **(95% CI), mL**	***P*-value**
Deep white matter hyperintensity grade	11 (2–20)	0.018	5 (1–10)	0.021	13 (−2–27)	0.089
Periventricular white matter hyperintensity grade	−5 (−13–2)	0.174	−1 (−4–2)	0.445	−3 (−9–2)	0.209
Enlarged perivascular spaces scale	1 (−1–2)	0.366	1 (0–2)	0.174	0 (-1–2)	0.602
Presence of old lacune	5 (−12–21)	0.574	7 (−14–29)	0.520	3 (−27–33)	0.850

*Pseudo-R^2^ values for this model at 25th, 50th, and 75th percentile were 0.06, 0.34, and 0.39, respectively*.

### Functional Outcome With Brain Volume Change

Brain volume change was associated with ordinal change on the mRS at 90 days (per 10 ml, odds ratio [*OR*] 1.085, 95% *CI* 1.031–1.141) but the association was not present when adjusting for age, baseline diffusion lesion volume, and NIHSS score (*OR* 1.044, 95% *CI* 0.990–1.010). In this model age, baseline diffusion lesion volume and NIHSS score were associated with outcome.

## Discussion

We found that global brain volume changes are heterogenous after stroke. Brain volume loss occurs over the month after stroke and is associated with age and deep WM disease. We did not find evidence that more severe strokes lead to increased early tissue loss.

There are only a few longitudinal studies of the time course of global brain volume change after non-lacunar ischemic stroke, and most have focused on more chronic stages after stroke. Global atrophy occurred at a 3-fold higher rate in cerebral autosomal dominant arteriopathy with subcortical infarcts and lacunes (CADASIL) patients compared with controls, independent from WM disease (16). Patients after minor stroke experienced higher whole-brain atrophy rates than healthy controls over a 3-year period (17). The whole-brain atrophy rate outside the frank lesion in the chronic poststroke brain occurred at a 0.95% per year of total brain volume (7). Regional changes in the brain volume after stroke have been found with declines in the volumes of the hippocampus and thalamus reported in the first 3 months after stroke (4). One recent study focusing on early atrophy, found a decrease in contralateral brain volume in a cohort of 50 patients of 14 ml after a mean of 3 months (18). We found a decrease in global volume of a larger magnitude occurring already within the first month after stroke. Interestingly in both studies, there were also patients with an increase in global brain volume. This probably reflects ongoing brain edema, hemorrhagic transformation, and measurement error. We found no evidence of large ischemic lesion growth in those with brain volume increase between baseline and follow-up.

The mechanisms of brain tissue loss over time are poorly understood. They may reflect tissue loss in the stroke area itself, (7) widespread WM fiber degeneration, (19) remote neuronal degeneration due to disconnection, (20) accelerated brain aging, (21) or neuroinflammation (22).

Adjusting for baseline brain volume, age was associated with larger brain volume loss in our study, corresponding to previous studies that indicate that brain atrophy accelerates with the increasing age (23–25). Our reported rates are, however, higher than those reported in aging. Brain volume peaks in the third and fourth decades and then declines at an increasing rate: for example, in octogenarians, total brain volumes decline on average by 0.64% per year (26, 27). Our findings may suggest that the occurrence of stroke accelerates an already pre-existent atrophic process, especially in the setting of vascular risk factors leading to the incident stroke, but this would ideally require a direct comparison with a non-stroke population and a close monitoring of risk factor modifying therapy.

We hypothesized that the tissue loss would be more pronounced in patients with more severe strokes but found little evidence for this. The NIHSS was weakly associated with larger brain volume decrease only in those patients with the largest brain volume change. Baseline and 30-day infarct volumes were not associated with larger brain volume change. We suspect that persistent edema within these lesions or the presence of hemorrhagic transformation may have obscured some of the atrophy occurring in other brain areas and the tissue loss within the ischemic lesion.

There is a strong relationship between cerebral atrophy and the presence of cerebral small vessel disease (6, 28). We therefore hypothesized that imaging manifestations of small vessel disease would increase volume loss after stroke. We found a possible association between increasing degrees of DWM with atrophy, but this was mostly pronounced in patients with smaller brain volume change over time. Our findings may not be representative as there were few patients with old lacunes on MRI in this study and acute lacunar strokes were excluded from the trial. Future studies should use semi-automated software to assess WMH volume to better confirm this association.

We did not observe a relationship between 30-day serial brain tissue loss and clinical outcome assessed using the mRS at 90 days. Atrophy has been linked to cognitive decline and memory loss which are not directly assessed by the mRS. Further studies should evaluate the relationship among acute brain volume loss and (longer term) cognitive impairment and other outcomes, such as fatigue.

This study has limitations. The observed brain volume changes were often small and may be related to technical problems, such as the use of clinical images with poor image resolution. Therefore, our findings need to be replicated. The imaging quality was variable among different centers and follow up scans were sometimes done on different machines. The recruitment of patients across multiple sites using multiple scanners is another source of variability. The intrasubject variability of brain volume measurements, reflecting the consequences of measurement error, and short-term biological fluctuations due to hydration and hormonal status has been reported to be 0.25% of total brain volume in healthy individuals assessed two times within 1 month (27, 29). We can assume that part of the reported changes reflects this variability. The use of 2D T2 weighted sequences of low resolution, rather than high resolution isotropic 3D T1 sequences to measure brain volume is also a limitation of this study. This explains why we are not able to assess localized atrophy and assess whether the atrophy is more pronounced in WM or GM. Lesion volume changes were estimated from subtracting lesion volumes from subacute DW images and 30d FLAIR images, with different voxel sizes and this may have introduced measurement error. Semiautomated measurement of WMH volume would have been preferable. No information on cognitive status was obtained.

## Conclusions

We found brain volume shrinkage after acute ischemic stroke occurring within a month associated mostly with age and the presence of DWMHs. Further studies should look at a possible link with inflammation and the location of the brain volume changes and its relationship with cognitive and other outcomes.

## Data Availability Statement

The original contributions presented in the study are included in the article/Supplementary Material, further inquiries can be directed to the corresponding author.

## Ethics Statement

The studies involving human participants were reviewed and approved by the Ethics Committees at the participating Trial Sites, the full list is available in the supplementary material. The patients/participants provided their written informed consent to participate in this study.

## Author Contributions

NB: data collection and drafting manuscript. LC: statistical analysis and revision of manuscript for important intellectual content. RL, AW, AC, GW, MG, BN, ER, AB, MK, and JF: data collection and revision of manuscript for important intellectual content. RL: obtained funding for original study, data collection, and revision of manuscript for important intellectual content. VT: study concept, drafting manuscript, and data collection. All authors contributed to the article and approved the submitted version.

## Funding

The study was supported by the China National Scholarship fund. The Florey Institute of Neuroscience and Mental Health acknowledges the strong support from the Victorian Government and in particular the funding from the Operational Infrastructure Support Grant. RL is a Senior Clinical Investigator of FWO Flanders and is supported by grants from the European Union.

## Conflict of Interest

RL was employed by Sygnis GmBh, the funder of the AXIS-2 study. Sygnis GmBh was not involved in the current study design, collection, analysis, interpretation of data, the writing of this article or the decision to submit it for publication. The remaining authors declare that the research was conducted in the absence of any commercial or financial relationships that could be construed as a potential conflict of interest.

## Publisher's Note

All claims expressed in this article are solely those of the authors and do not necessarily represent those of their affiliated organizations, or those of the publisher, the editors and the reviewers. Any product that may be evaluated in this article, or claim that may be made by its manufacturer, is not guaranteed or endorsed by the publisher.

## Abbreviations

BG: basal ganglion
CADASIL: cerebral autosomal dominant arteriopathy with subcortical infarcts and lacunes
CI: confidence interval
CSF: cerebrospinal fluid
CSO: centrum semiovale
DWI: diffusion weighted imaging
DWMHs: deep white matter hyperintensities
EPVSs: enlarged perivascular spaces
FLAIR: fluid attenuation inversion recovery
G-CSF: granulocyte colony-stimulating factor
GM: gray matter
IQR: interquartile range
MB: middle brain
MRI: magnetic resonance imaging
mRS: modified Rankin scale
NIHSS: National Institutes of Health Stroke Scale
PWMHs: periventricular white matter hyperintensities
rt-PA: recombinant tissue plasminogen activator
SVD: small vessel disease
WM: white matter
WMH: white matter hyperintensities.
