# Supplementary material for: Early Brain Volume Changes After Stroke: Subgroup Analysis From the AXIS-2 Trial
Source: Front Neurol. 2022 Jan 28;12:747343. doi: 10.3389/fneur.2021.747343 (PMC8832974; doi:10.3389/fneur.2021.747343)
Supplement: Supplementary file 1 [file Data_Sheet_1.docx]

| Uniklinik RWTH Aachen | Aachen | Germany |
| --- | --- | --- |
| Klinikum Altenburger Land | Altenburg | Germany |
| Hospital Clinic de Barcelona | Barcelona | Spain |
| Hospital de la Santa Creu i Sant Pau | Barcelona | Spain |
| Charite Hospital | Berlin | Germany |
| Medical University of Bialystok | Białystok | Poland |
| Klinikum Bielefeld | Bielefeld | Germany |
| Neurologická klinika LF UK | Bratislava | Slovakia |
| Comenius University | Bratislava | Slovakia |
| Klinikum Bremen-Mitte | Bremen | Germany |
| AZ Sint Jan | Bruges | Belgium |
| Cliniques Universitaires Saint-Luc | Brussels | Belgium |
| Klinikum Chemnitz | Chemnitz | Germany |
| Kliniken der Heinrich-Heine-Universität Düsseldorf | Düsseldorf | Germany |
| Universitätsklinikum Erlangen | Erlangen | Germany |
| Universitätsklinikum Essen | Essen | Germany |
| Erich-Lexer-Klinik | Freiburg im Breisgau | Germany |
| Universitair Hospitaal Ghent | Ghent | Belgium |
| Hospital Universitari de Girona Dr. Josep Trueta | Girona | Spain |
| Universitätsklinikum Halle | Halle | Germany |
| Universitätsklinikum Eppendorf | Hamburg | Germany |
| Universitätsklinikum Heidelberg | Heidelberg | Germany |
| Universitätsklinikum Jena | Jena | Germany |
| Universitätsklinikum Leipzig | Leipzig | Germany |
| Universitair Hospitaal Leuven | Leuven | Belgium |
| Kepler Universitätsklinikum | Linz | Austria |
| Wagner-Jauregg Klinikum | Linz | Austria |
| Klinikum Ludwigshafen | Ludwigshafen | Germany |
| Hospital Ramón y Cajal | Madrid | Spain |
| Universidad Autónoma de Madrid | Madrid | Spain |
| Universitätsklinikum Gießen und Marburg (UKGM) | Marburg | Germany |
| Helios Klinikum | Munich | Germany |
| Universitätsklinikum Münster | Münster | Germany |
| Fakultná Nemocnica Nitra | Nitra | Slovakia |
| University Hospital Olomouc | Olomouc | Czech Republic |
| Klinikum Osnabrück | Osnabrück | Germany |
| Městská nemocnice Ostrava | Ostrava | Czech Republic |
| Ostrava University Hospital | Ostrava | Czech Republic |
| Fakultní nemocnice Plzeň | Pilsen | Czech Republic |
| Fakultní nemocnice v Motole | Prague | Czech Republic |
| Fakultní nemocnice v Praze | Prague | Czech Republic |
| Hospital Universitario Virgen del Rocío | Seville | Spain |
| Asklepios Fachklinikum Teupitz | Teupitz | Germany |
| Fakultná Nemocnica Trnava | Trnava | Slovakia |
| Universitätsklinikum Tübingen | Tübingen | Germany |
| Universitätsklinikum Ulm | Ulm | Germany |
| Hospital Clínico Universitario de Valencia | Valencia | Spain |
| Wiener Krankenanstaltenverbund | Vienna | Austria |
| Kliniki Neurologicznej Instytutu Psychiatrii | Warsaw | Poland |
| Institute of Psychiatry and Neurology | Warsaw | Poland |
